# Draft Genome Sequence of Pantoea agglomerans CPHN2, a Potential Plant-Growth-Promoting Endophyte

**DOI:** 10.1128/mra.00192-22

**Published:** 2022-07-21

**Authors:** Pradeep Kumar, Varsha Chauhan, Amita Suneja Dang, Ajit Kumar, Pooja Suneja

**Affiliations:** a Department of Microbiology, M.D. University, Rohtak, India; b Centre for Medical Biotechnology, M.D. University, Rohtak, India; c Centre for Bioinformatics, M.D. University, Rohtak, India; University of Arizona

## Abstract

Here, we report the draft genome sequence of Pantoea agglomerans CPHN2, an endophyte isolated from nodules of Cicer arietinum (Chickpea) from Hisar, Haryana, India. The genome was 4,839,532 bp and exhibited a GC content of 55.2% and 4,508 genes with 4,468 coding sequences, 1 rRNA, 71 tRNAs, and 1 CRISPR.

## ANNOUNCEMENT

Members of *Pantoea*, a genus of Gram-negative bacteria, have been isolated from diverse habitats and been found to exhibit an association with a variety of hosts, such as plants, animals, and humans. Many species of this genus are associated with plants, either on their surfaces or as endophytes. Pantoea agglomerans, one of the most common species associated with plants, is able to fix atmospheric nitrogen and possess multiple plant-growth-promoting (multi-PGP) traits. Numerous studies have reported the entophytic occurrence of *P. agglomerans* in various crops, such as rice (1), chickpea (2), sugarcane (3), sweet potato (4), and wheat (5).

*P. agglomerans* CPHN2, a nonrhizobial endophyte, was isolated from nodules of Cicer arietinum (chickpea) from Hisar, Haryana, India (29.25°N, 76.05°E), with a motive to access its ability to promote plant growth. Healthy nodules of *C. arietinum* were detached from the plants and surface sterilized (6). The uncrushed sterilized nodules were placed on tryptone soya agar (TSA) plates as a control to check the efficacy of surface sterilization. Macerated samples were streaked onto TSA plates and examined for the growth of endophytic bacteria after an incubation (28 ± 2°C) of 3 days. The colonies were selected and purified. The genomic DNA was isolated using a modified cetyltrimethylammonium bromide (CTAB) protocol (7) and amplified using 16S rRNA universal primers (1541R and 8F) (8). The amplicon was sequenced, a similarity analysis was performed at the BLAST server, and the sequence was submitted to GenBank (MH298522). The isolate was observed to possess plant-growth-promoting traits, such as the solubilization of phosphate and the production of ammonia, siderophore, and a large amount of indole-3-acetic acid (9). The presence of these prominent plant-growth-promoting traits encouraged us to go for whole-genome sequencing of this strain which further confirmed it as Pantoea agglomerans.

A paired-end (PE) sequencing library was prepared from the DNA sample using the Illumina TruSeq nano DNA library prep kit. The PE Illumina library was loaded into the NextSeq 500 instrument for cluster generation and sequencing. Paired-end sequencing on the NextSeq 500 instrument helps in reading template fragments in both forward and backward orientations. Whole-genome sequencing (Illumina NextSeq 500, 2 × 150 bp) yielded 2,898,434,450 reads representing ~100× genome coverage. Fastp v0.201 (10), Shovill v1.0.4 (11), and Prokka v1.14.5 (12) tools of the Galaxy server were used for genome analysis and annotation. Fastp v0.201 was used to filter and trim the final assembly, and the genome sequence was assembled with Shovill v1.0.4. Default parameters were used for the software. After the filtering and trimming steps, a total of 9,731,611 paired-end reads were obtained. Shovill combined the reads into 32 contigs, resulting in a total assembly size of 4,839,532 bp and an *N*_50_ value of 558,390 bp. Prokka v1.14.5 was used for the annotation, yielding 4,468 coding sequences. The genome was also observed to carry 1 rRNA gene, 71 tRNA genes, and 1 CRISPR gene. The GC content of the genome was found to be 55.2%. The genome was annotated automatically using Galaxy (https://usegalaxy.org/) for a further comparative analysis and genome annotation and RAST (https://rast.nmpdr.org/) for a better graphical analysis (13).

The reported draft genome sequence of *P. agglomerans* CPHN2 will help us improve strain-specific fingerprinting, which is essential for the registration and protection of commercial bioinoculant products. Also, this draft genome sequence of *P. agglomerans* CPHN2 will provide a platform for elucidating various plant-growth-promoting mechanisms and their regulation, which can be used to further improve the practical and commercial value of the strain by optimization of formulation technology, performance, and efficacy of the *P. agglomerans* CPHN2 strain.

### Data availability.

The *P. agglomerans* CPHN2 whole-genome sequence has been deposited at GenBank under the accession no. CP098412 and CP098413 for plasmids and CP098414 for the chromosome and annotated by the prokaryotic genome annotation pipeline (PGAP) (14). The BioProject accession no. is PRJNA811747 (Sequence Read Archive accession no. SRR18189611).
